# The gut-lung axis: pathological crosstalk and inter-organ communication in chronic obstructive pulmonary disease and inflammatory bowel disease

**DOI:** 10.3389/fimmu.2026.1796944

**Published:** 2026-04-22

**Authors:** Xinying Liu, Shuo Yang, Yingying Yan, Lin Zhang, Xiaokun Yang, Li Liu

**Affiliations:** 1 First Teaching Hospital of Tianjin University of Traditional Chinese Medicine, Tianjin, China; 2 National Clinical Research Center for Chinese Medicine, Tianjin, China

**Keywords:** common mucosal immune system, gut-lung axis, inflammatory bowel diseases, lymphocyte homing, pulmonary disease, chronic obstructive

## Abstract

The significant bidirectional comorbidity risk and extensive subclinical involvement observed between chronic obstructive pulmonary disease (COPD) and inflammatory bowel disease (IBD) underscore the pivotal role of the “gut-lung axis” in cross-organ pathological crosstalk. Here, we comprehensively review the molecular and immunological mechanisms driving this comorbidity. Genome-wide association studies (GWAS) have substantiated genetic pleiotropy that underpins a shared susceptibility to mucosal defense deficits. The “common mucosal immune system” (CMIS), rooted in embryonic homology, constitutes the anatomical basis for this pathological interplay, wherein aberrant immune cell homing, Th17/Treg imbalance, and the cross-organ trafficking of innate lymphoid cells (ILCs) mediate the distal dissemination of inflammation. Furthermore, gut dysbiosis-induced depletion of short-chain fatty acids (SCFAs), acting in concert with systemic hypoxia and the IL-23/IL-17 axis, potentiates synergistic injury to the gut-lung barriers. We highlight the reciprocal, bidirectional causality of this “hypoxic loop” and its testable mechanistic predictions for barrier dysfunction. Furthermore, we evaluate pharmacological evidence from drug repositioning, alongside a critical examination of the “hidden axis” of clinical therapies as profound iatrogenic confounders. Elucidating these mechanisms is critical for establishing systemic diagnostic and therapeutic strategies; interventions targeting shared molecular targets and the microbiota hold promise for achieving a simultaneous treatment approach for these distinct pathologies.

## 1 Introduction

Chronic obstructive pulmonary disease (COPD) and inflammatory bowel disease (IBD) represent a significant global burden of chronic inflammatory disorders. Historically, respiratory and gastrointestinal pathologies have been compartmentalized as distinct clinical entities. However, accumulating clinical and epidemiological evidence underscores a significant comorbid association between the two (1, 2). Specifically, patients with COPD frequently manifest gastrointestinal dysfunction and metabolic dysregulation, whereas individuals with IBD often exhibit subclinical pulmonary functional impairment and airway inflammation, particularly during periods of disease activity (3, 4). This observed concordance in susceptibility, disease trajectory, and therapeutic response suggests that these pathologies are not isolated phenomena but are likely driven by shared underlying pathophysiological mechanisms.

The biological underpinnings of this cross-organ interrelationship are rooted in their shared anatomical and developmental homology. Originating from the embryonic foregut, the lungs and the intestine not only possess histologically similar columnar epithelium and mucus layers but also share the “Common Mucosal Immune System” (CMIS) (5, 6). Predicated on this physiological foundation, the concept of the “gut-lung axis” has emerged as a pivotal theoretical framework for elucidating cross-organ mucosal immune crosstalk (7, 8). Rather than a solitary anatomical conduit, the gut-lung axis represents an intricate, bidirectional regulatory network encompassing the microbiota, immune cell trafficking, and metabolic byproducts (9, 10). Under homeostatic conditions, this system orchestrates synergistic mucosal immune tolerance; conversely, in pathological states, localized inflammatory signals can propagate through this axis to trigger distal immune activation, instigating a cross-organ inflammatory cascade that culminates in multi-organ involvement (11, 12).

While extant epidemiological evidence has firmly delineated the bidirectional comorbidity risk between COPD and IBD, the deep-seated mechanisms driving this association remain labyrinthine. A confluence of multifarious factors, encompassing overlapping genetic susceptibility, aberrant cross-organ immune cell homing, gut dysbiosis, and a systemic hypoxic microenvironment, coalesce to construct an intricate network of pathological interaction. Consequently, a profound interrogation of these trans-systemic pathological nexuses is imperative; such efforts will not only facilitate the dismantling of traditional siloed diagnostic and treatment paradigms but also unveil novel therapeutic avenues for the management of refractory comorbid cases in clinical settings.

Therefore, we aim to provide a systematic synthesis of recent advances characterizing the epidemiology, shared genetic architecture, and pathophysiological mechanisms linking COPD and IBD. To provide a critical hierarchy of this data, **Table 1** maps each primary proposed mechanism to the strongest available level of evidence, distinguishing between human (associational/biomarker) and experimental (causal) data. Specifically, we dissect the aberrant ectopic migration of immune cells, metabolic modulation by the gut microbiota, and cross-system crosstalk of pivotal molecular signaling pathways. Ultimately, this comprehensive analysis seeks to furnish a robust theoretical foundation for developing synergistic diagnostic strategies and precision targeted interventions for these concurrent chronic mucosal inflammatory diseases.

**Table 1:** Mapping proposed gut-lung axis mechanisms to current evidence levels.

Mechanism	Animal/experimental evidence	Human evidence
CMIS & Lymphocyte Trafficking (CCR9/CCL25)	Pulmonary DCs directly imprint gut-homing integrins (α4β7) on T cells; T cells migrate to inflamed gut tissue.	Expression of homing markers identified in peripheral blood; predominantly associative evidence linking disease states.
Th17/Treg Imbalance & IL-23/IL-17 Axis	SCFA depletion impairs Treg maintenance; IL-23 directly drives Th17 lung structural damage.	GWAS robustly links *IL23R* to IBD; Th17-related cytokines are elevated in both COPD and IBD patient mucosa.
Microbial Metabolites (SCFA Depletion)	Butyrate administration directly reduces ILC2 airway inflammation and promotes localized Treg differentiation.	Reduced *Lachnospiraceae* in COPD patient stool; altered systemic SCFA profiles correlate with declined lung function.
The “Hypoxic Loop”	Chronic hypoxia drives HIF-2α accumulation, resulting in barrier leakage and LPS translocation in experimental models.	Elevated LPS-binding protein and Zonulin in COPD plasma; systemic VEGF levels correlate with IBD severity.

CCL25, CC chemokine ligand 25; CCR9, CC chemokine receptor 9; CMIS, common mucosal immune system; COPD, chronic obstructive pulmonary disease; DCs, dendritic cells; GWAS, genome-wide association studies; HIF-2α, hypoxia-inducible factor-2 alpha; IBD, inflammatory bowel disease; IL-17, interleukin-17; IL-23, interleukin-23; IL23R, interleukin-23 receptor; ILC2, group 2 innate lymphoid cell; LPS, lipopolysaccharide; SCFA, short-chain fatty acid; Th17, T helper 17 cell; Treg, regulatory T cell; VEGF, vascular endothelial growth factor.

## 2 Convergent epidemiology and the genetic architecture of comorbidity

### 2.1 Bidirectional comorbidity risk between COPD and IBD

Recent large-scale clinical cohort studies have substantiated a significant bidirectional association between IBD and COPD. Notably, this comorbidity is frequently concomitant with extensive cross-system subclinical abnormalities, thereby implicating shared underlying pathophysiological mechanisms between these distinct inflammatory entities (13–15).

Retrospective cohort studies have demonstrated that patients with IBD exhibit an approximately 30% elevated risk of developing COPD compared to the general population (16). Corroborating these findings, prospective cohort data similarly highlight a heightened susceptibility to COPD within the IBD cohort (17, 18). Crucially, this risk profile extends beyond mere incidence to profoundly impact disease prognosis. Comorbid IBD is associated with a significant amplification in the frequency of acute COPD exacerbations and all-cause mortality, thereby perpetuating a vicious “lung-gut” cycle of pathological interaction (19).

Crucially, this risk profile is characterized by distinct bidirectionality. Patients with COPD demonstrate a 60% increased likelihood of a subsequent IBD diagnosis (18, 20). Furthermore, IBD susceptibility correlates positively with the severity of COPD, implying that more advanced pulmonary pathology predisposes patients to greater intestinal involvement. Beyond individual comorbidity, a significantly elevated risk of IBD is observed among first-degree relatives of COPD patients, a familial clustering pattern that strongly implicates a shared genetic susceptibility underlying both conditions (21). Additionally, early-life environmental exposures, such as cigarette smoke (CS) and antibiotic overuse, may serve as shared triggers, playing a pivotal role in the etiology and initiation of these distinct pathologies.

Furthermore, the pervasive subclinical organ involvement observed across both IBD and COPD cohorts furnishes a direct pathophysiological rationale for this comorbidity. On the pulmonary front, occult pulmonary pathologies are frequently detected in IBD patients devoid of overt respiratory symptoms. Approximately 43.58% of IBD patients exhibit pulmonary function anomalies, predominantly characterized by small airway dysfunction. Correspondingly, high-resolution computed tomography frequently reveals bronchial wall thickening and mosaic attenuation. Notably, the magnitude of this pulmonary involvement correlates positively with IBD disease activity indices (2, 22, 23). Conversely, patients with COPD frequently manifest systemic dysregulation, most notably compromised intestinal barrier function (clinically termed “leaky gut”). The severity of this intestinal mucosal injury closely parallels the clinical activity and progression of COPD (1, 4).

### 2.2 Genetic pleiotropy and shared molecular networks

Both IBD and COPD are classified as complex polygenic disorders. Genome-wide association studies (GWAS) have unveiled significant genetic pleiotropy between these conditions, where a single genetic variant influences multiple distinct traits, thereby establishing a molecular foundation for their comorbidity. A prime exemplar is NOD2, a pivotal susceptibility gene for Crohn’s disease, which encodes an intracellular receptor responsible for recognizing bacterial muramyl dipeptide and subsequently activating the nuclear factor kappa B (NF-κB) signaling pathway (24). Research has confirmed that NOD2 variants not only drive intestinal inflammation but also impair pathogen recognition capabilities in pulmonary macrophages (25–27). This defect in cross-organ innate immune recognition mechanisms compromises the mucosal defense barrier, rendering both the lungs and the gut susceptible to dysbiosis (28). Furthermore, cross-trait GWAS analyses encompassing diverse immune-mediated diseases, including COPD and IBD, have identified 57 shared risk loci, with significant enrichment observed in the 17q12 and 16p11.2 chromosomal regions (29). Subsequent fine-mapping analysis has pinpointed 162 pleiotropic genes that modulate susceptibility across a spectrum of autoimmune and inflammatory pathologies.

Studies have revealed that the genetic loci shared by COPD and IBD are significantly enriched within mucosal immune regulatory pathways, encompassing T-cell activation, T-cell differentiation, and Type II interferon responses (30). Specifically, variants in ORMDL3 and GSDMB have been implicated in compromising epithelial integrity and reparative capacity, serving as a putative ‘genetic bridge’ linking epithelial fragility across the gut-lung axis (31). Mechanistically, MAPK3 modulates cytokine secretion via the mitogen-activated protein kinase signaling pathway, while genes such as BACH2 are crucial for the differentiation and activation of T cells (32, 33). However, these genetic associations do not function as deterministic drivers. Collectively, these loci constitute an intricate susceptibility network that, rather than strictly dictating disease onset, likely lowers the threshold for mucosal inflammation. This genetic background presumably interacts with environmental triggers, such as cigarette smoke and dietary factors, through epigenetic modifications, thereby shaping an individual’s predisposition to concurrent mucosal inflammatory pathologies (30, 34).

### 2.3 Pharmacological evidence revealing a shared pathological network

Beyond epidemiological clustering and genetic pleiotropy, clinical pharmacology provides a mechanistic framework that illuminates the pulmonary-intestinal pathological unity. The phenomenon of “drug repositioning”, where a therapy developed for one mucosal compartment proves effective in another—implies a deep-seated coupling in their pathogenesis.

Phosphodiesterase-4 (PDE4) inhibitors represent a classic class of transmucosal anti-inflammatory agents. Roflumilast, the first selective PDE4 inhibitor approved for COPD, effectively suppresses neutrophil-mediated inflammation by inhibiting cyclic adenosine monophosphate degradation (35, 36). Building on this, the dual PDE3/PDE4 inhibitor ensifentrine combines bronchodilatory and anti-inflammatory effects, further improving patients’ lung function and quality of life (37, 38). Notably, the action of this drug class is not confined to the lungs. Preclinical studies demonstrate that various PDE4 inhibitors, including roflumilast, rolipram, tetomilast, and apremilast, significantly mitigate damage in DSS-induced colitis. Their protective mechanisms are multidimensional. These include anti-inflammatory activity, whereby they reduce reduce tumor necrosis factor-alpha (TNF-α), nitric oxide, and myeloperoxidase activity to inhibit neutrophil infiltration; barrier repair, mediated through the downregulation of inducible nitric oxide synthase to decrease intestinal mucosal permeability; and anti-fibrotic effects, which involve reducing collagen deposition to block the progression from chronic inflammation to fibrosis (39).

Sphingosine-1-phosphate (S1P) receptor modulators link pulmonary and intestinal pathology through dual perspectives of immune cell migration and tissue barrier integrity. Newer agents like ozanimod and etrasimod, used in IBD, induce S1P receptor internalization, sequestering lymphocytes within lymph nodes and blocking their pathological migration to the intestinal mucosa (40). In the lungs, S1P signaling is crucial for maintaining alveolar-capillary barrier integrity; it promotes endothelial cytoskeletal rearrangement and strengthens tight and adherens junctions, thereby mitigating pulmonary leakage and acute lung injury. Although current indications for these drugs are centered on IBD, their potential as “endothelial barrier fortifiers” in COPD has garnered attention. Future development of inhaled formulations could enable localized treatment of respiratory exudative inflammation while exerting systemic immunomodulatory effects via the gut-lung axis (41).

Janus kinase (JAK) inhibitors offer another perspective for cross-mucosal immune intervention. The highly selective JAK1 inhibitor upadacitinib, owing to its effective blockade of pathways such as IL-6 and its favorable safety profile, has been successfully deployed in Crohn’s disease and ulcerative colitis (42, 43). In the respiratory realm, the efficacy demonstrated by tofacitinib in rheumatoid arthritis-associated interstitial lung disease suggests a regulatory role for the JAK pathway in pulmonary fibrosis and immune dysregulation (44). Given that advanced COPD often exhibits autoimmune-like features and interstitial remodeling, JAK inhibitors may be applicable to specific inflammatory-fibrotic phenotypes (45, 46). However, their translation faces safety challenges: in patients aged 50 and above with a history of smoking, who represent the core COPD demographic, JAK inhibitors significantly increase the risk of major adverse cardiovascular events, venous thromboembolism, and lung cancer (47). Consequently, the application of these agents in COPD requires cautious risk-benefit assessment and stringent patient subgroup selection.

However, it is crucial to recognize that PDE4 inhibitors, S1P modulators, and JAK inhibitors are fundamentally systemic immunomodulators. Their cross-organ benefit does not necessarily validate an exclusive “gut-lung axis”; it may simply reflect shared, parallel systemic inflammatory pathways. To definitively prove a gut-lung axis via pharmacology, future research must identify true “axis-specific” evidence. This would entail demonstrating that improvements in lung function are directly mediated by pharmacological restoration of gut permeability, normalization of short-chain fatty acids (SCFAs) profiles, or altered organ-specific homing signatures, rather than a generalized dampening of systemic immunity.

## 3 Anatomical and immunological foundations of the CMIS

### 3.1 Embryonic homology and structural conservation of the lung-gut barrier

Developmentally, both the lungs and the intestine originate from the embryonic foregut endoderm. This shared lineage dictates a high degree of histological similarity between the two organs. Both the respiratory and gastrointestinal tracts are lined by an epithelial cell layer that functions as a critical physical barrier, overlaid by a mucus layer secreted by goblet cells. Together, these structures constitute the first line of defense against exogenous antigens (48–50). Crucially, this structural homology extends beyond mere morphology to the expression of functional proteins; both tissues express analogous pattern recognition receptors (PRRs), such as Toll-like receptors (TLRs) and Nod-like receptors (NLRs), to detect microbial signals (5, 51–53). This anatomical foundation implies that, in response to environmental stimuli, both systems frequently initiate convergent signal transduction pathways.

### 3.2 Physiological trafficking and pathological dysregulation of mucosal immune cells

The central tenet of the CMIS posits that immune cells, once sensitized to antigens at a specific mucosal inductive site, can migrate via the systemic circulation to exert effector functions at distal mucosal sites (54, 55). Consequently, dysfunction within the CMIS is postulated as a shared mechanism driving the rising prevalence of concurrent mucosal inflammatory pathologies, including IBD, asthma, and COPD.

Anatomically, both intestinal Peyer’s patches and pulmonary bronchus-associated lymphoid tissue harbor specialized Microfold cells (56). Devoid of typical microvilli, Microfold cells possess a unique capacity for transcytosis, allowing them to sample luminal antigens and translocate them to underlying dendritic cells (DCs) and lymphocytes. This antigen sampling process represents the critical rate-limiting step in the initiation of antigen-specific mucosal immune responses. Furthermore, sIgA serves as the principal effector molecule for systemic mucosal protection within the CMIS. Following induction in the intestinal mucosa, IgA-producing plasma cells are capable of migrating to the respiratory mucosa, where they secrete sIgA targeting the specific antigen originally encountered in the gut.

However, under pathological conditions, this protective mechanism may undergo maladaptation. The pro-inflammatory microenvironment generated by localized inflammation can systemically alter immune cell homing signatures. This perturbation significantly amplifies the risk of aberrant migration and infiltration of inflammatory subsets, such as effector T cells, into distal mucosal organs via shared CMIS pathways, thereby propagating inflammation across the gut-lung axis (57, 58).

**Figure 1:**
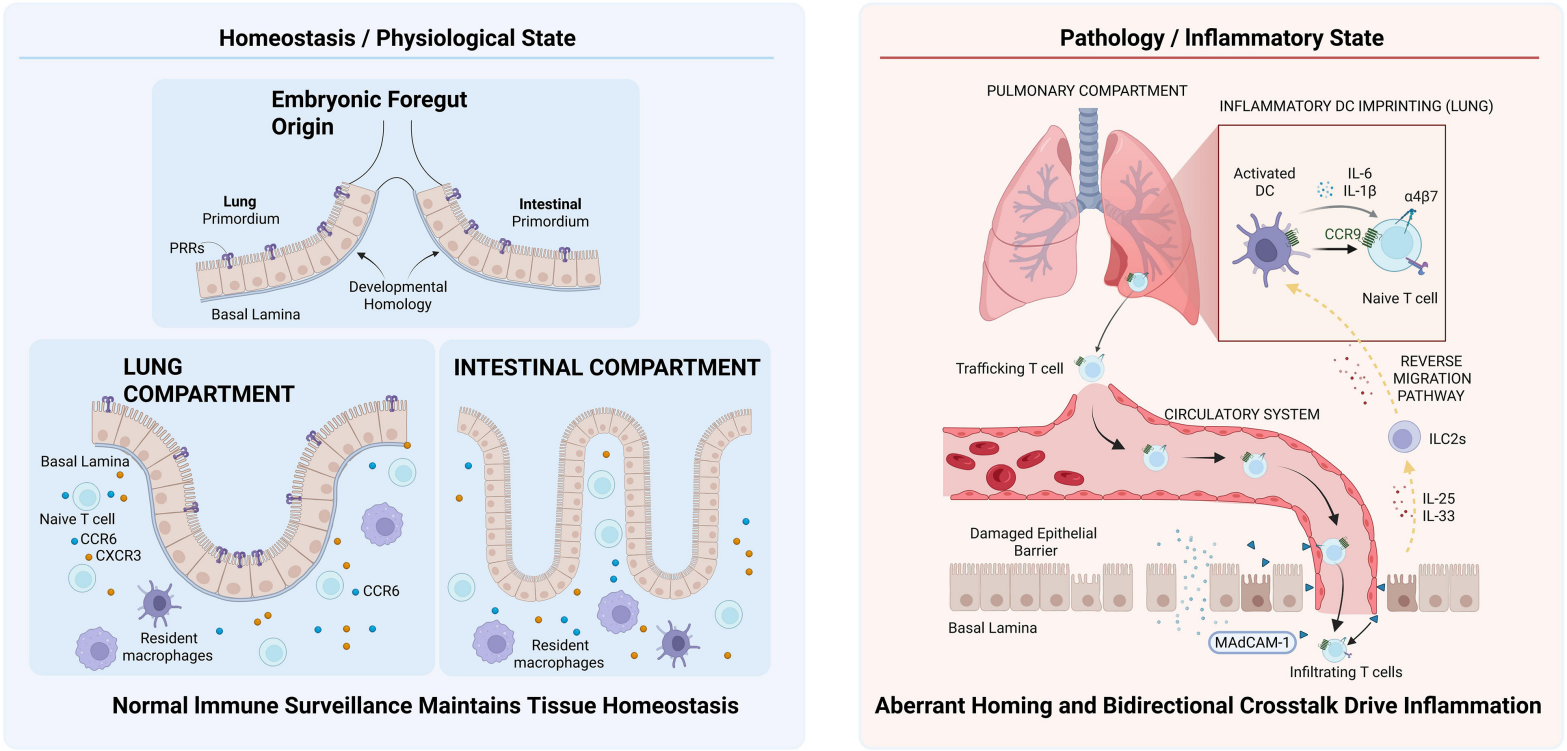
The’gut-lung axis’ architecture and CMIS crosstalk. Developmentally, the lungs and intestine originate from the embryonic foregut, sharing homologous columnar epithelium and pattern recognition receptors (PRRs). Under pathological conditions (e.g., COPD), pulmonary dendritic cells (DCs) imprint CD4+ T cells with gut-homing receptors (CCR9 and α4β7). These cells migrate via the circulation to the intestine, guided by CCL25 and MAdCAM-1, propagating inflammation. Innate Lymphoid Cells (ILCs) exhibit inter-organ plasticity: gut-derived ILC2s migrate to the lungs in response to inflammatory alarmins (IL-25/IL-33).

Collectively, these developmental and immunological parallels constitute the structural basis of the gut–lung axis. Under physiological conditions, the CMIS orchestrates a balanced surveillance network; however, in pathological states like COPD, this shared infrastructure is hijacked, facilitating the aberrant ectopic migration of immune cells and inflammatory mediators between the lungs and the intestine (**Figure 1**).

## 4 Cellular drivers of cross-organ crosstalk

### 4.1 The Initiator of mucosal immune dysregulation

Gut dysbiosis in COPD patients manifests not merely as a reduction in overall community diversity but, more critically, as disease-specific taxonomic alterations (59). Multi-omics association analyses have substantiated significant correlations between the abundance of specific microbial taxa and clinical disease phenotypes, such as inflammation and emphysema (60). In CS-induced murine models of COPD, significant perturbations in the abundance of families such as *Muribaculaceae* and *Desulfovibrionaceae* have been observed (61). Notably, the depletion of *Lachnospiraceae* is directly linked to impaired host immunomodulatory functions, suggesting a putative protective role for this family in maintaining “gut-lung” immune homeostasis (62).

Functional profiling reveals a marked downregulation of pathways involved in glucose and starch metabolism within the COPD-associated microbiome. This diminished capacity to process complex carbohydrates likely contributes to a paucity of biosynthetic substrates for SCFAs and a consequent decline in their production, thereby attenuating the gut-mediated distal suppression of pulmonary inflammation (63).

Of particular note is the nuance regarding the immunomodulatory mechanisms of specific commensals: not all Lipopolysaccharides (LPS) derived from Gram-negative bacteria possess potent pro-inflammatory properties. Research identifies that *Parabacteroides goldsteinii* exhibits distinct anti-inflammatory activity in COPD models (64). Mechanistically, LPS extracted from this bacterium functions by antagonizing TLR4 signaling, thereby ameliorating both pulmonary and intestinal inflammation and correcting metabolic dysregulation. This finding elucidates a novel mechanism by which commensals participate in host immune regulation, challenging the dogma of LPS as a pro-inflammatory mediator. This dysbiotic microenvironment serves as a crucial instigator for the subsequent immune dysregulation described below.

### 4.2 Aberrant lymphocyte homing mediated by the CCR9/CCL25 axis

Lymphocyte homing is orchestrated by highly specific interactions between integrin-ligand pairs and chemokine-receptor axes. In the context of comorbid IBD and COPD, perturbation of this mechanism may contribute to the cross-organ ectopic infiltration of immune cells. Under physiological conditions, the CCL25-CCR9 axis directs lymphocyte homing specifically to the small intestine, a mechanism critical for the maintenance of intestinal immune homeostasis (65, 66).

However, under states of pulmonary inflammation, such as COPD or influenza infection, the pulmonary microenvironment undergoes significant remodeling. Research demonstrates that upon inflammatory stimulation, pulmonary DCs can induce the aberrant upregulation of CCR9 and integrin α4β7 on lung-derived CD4+ T cells. Subsequently, these “erroneously imprinted” effector T cells enter the systemic circulation and home to the intestine. There, they secrete pro-inflammatory cytokines, such as interferon-gamma (IFN-γ), thereby compromising the intestinal mucosal barrier and potentially instigating secondary dysbiosis (67).

Integrin α4β7 typically binds to the intestinal endothelial addressin mucosal vascular addressin cell adhesion molecule 1 (MAdCAM-1), mediating lymphocyte trafficking into the intestinal lamina propria (68). However, mechanistic studies reveal that in the presence of pulmonary inflammation, pulmonary DCs, driven by factors such as retinoic acid, can aberrantly induce the expression of these gut-homing receptors (CCR9 and α4β7) on lung-derived CD4+ T cells. Conversely, under pathological conditions, the pulmonary vascular endothelium may aberrantly express MAdCAM-1, thereby facilitating the infiltration of α4β7-expressing gut-derived lymphocytes into the lungs (69–71). This phenomenon of shared homing targets theoretically identifies a potent therapeutic avenue: the blockade of specific homing pathways could simultaneously alleviate dual gut-lung inflammation, offering molecular validation for therapeutic strategies centered on “treating pulmonary pathology via the gut” or vice versa.

### 4.3 T helper 17/Treg imbalance and the IL-23/IL-17 axis

The dynamic equilibrium between Th17 cells and regulatory T cells (Tregs) constitutes the linchpin of mucosal immune homeostasis. In the context of comorbid IBD and COPD, this equilibrium is skewed, manifesting as Th17-driven hyperinflammation concomitant with a deficiency in Treg-mediated immune tolerance (72, 73). This pathological skewing is critically sustained by the IL-23/IL-17 axis, a central conduit bridging innate and adaptive immunity (74, 75). Predominantly secreted by activated dendritic cells and macrophages, IL-23 is indispensable for the maintenance and expansion of Th17 cells. GWAS have unequivocally identified the IL-23R (IL-23 receptor) as a potent susceptibility locus for IBD, underscoring the genetic basis of this pathway (76). In disease progression, IL-23 drives Th17 cells to secrete pro-inflammatory cytokines, including IL-17A, IL-17F, and IL-22 (77). In IBD, this orchestrates neutrophilic infiltration and tissue injury (78). Analogously, in COPD, IL-17A serves as a pivotal mediator of airway neutrophilic inflammation and structural damage (79). At the transcriptional level, the overexpression of RORC (encoding RORγt), coupled with reciprocal inhibition of the Treg transcription factor FOXP3, synergistically promotes lineage skewing towards a Th17 phenotype (80).

This axis is also modulated by host-microbe interactions. On the pro-inflammatory side, segmented filamentous bacteria (SFB) in the gut induce intestinal epithelial cells to produce Serum Amyloid A, which acts on dendritic cells to potentiate Th17 differentiation via IL-23. Conversely, the gut microbiota exerts a decisive influence on Treg maintenance. SCFAs particularly butyrate produced by *Clostridium* clusters and *Bacteroides fragilis*, induce the synthesis of TGF-β1 by intestinal epithelial cells, thereby promoting Treg differentiation (9, 81). However, in COPD patients, the characteristic depletion of SCFA-producing bacteria attenuates these systemic Treg-inducing signals, undermining the negative feedback regulation of pulmonary inflammation. Reciprocally, pulmonary pathology can impact this gut equilibrium; excessive liberation of IL-22 triggered by pulmonary viral infections can stimulate intestinal expression of RegIIIγ via the systemic circulation. This antimicrobial peptide suppresses intestinal commensals, further exacerbating the Th17/Treg imbalance and unveiling a complex cross-organ cytokine regulatory network (82). Notably, specific probiotic interventions, such as *Lactobacillus rhamnosus*, have been shown to re-establish this balance by activating the TLR2 pathway and augmenting IL-10 production (83).

The significant therapeutic success of IL-23/IL-17 axis-targeting biologics in IBD lays the groundwork for precision medicine approaches aimed at “Th17-high” molecular subgroups of COPD.

### 4.4 Inter-organ trafficking of innate lymphoid cells

ILCs are strategically enriched at mucosal interfaces, functioning as a pivotal bridge between innate and adaptive immunity. Acting as rapid sentinels of environmental signals, they serve as immediate response hubs within the gut-lung axis interaction. Emerging evidence reveals that group 2 ILCs (ILC2s) residing in the gut and lungs possess remarkable inter-tissue migratory plasticity (84). Upon intestinal helminth infection or IL-25 stimulation, inflammatory ILC2s can mobilize, migrating via the mesenteric lymph nodes and systemic circulation to the lungs, where they contribute to pulmonary allergic inflammation or anti-helminth defense (85). This trafficking is governed by distinct chemotactic axes. The pulmonary IL-33/CXCL16 axis recruits natural ILC2s (nILC2s), whereas gut-derived iILC2 accumulation and migration depend on the IL-25/CCL25 axis (86, 87). A memory-like ILC2 subset (ml-ILC2s) has been identified that, upon CCR9/CCL25-mediated chemotaxis, resides long-term in the small intestinal lamina propria during disease remission and rapidly migrates back to the airways upon re-stimulation, contributing to asthma recurrence (88).

Furthermore, dysbiosis of the gut microbiota, characterized notably by an expansion of Proteobacteria, can elicit the host production of alarmins such as IL-33, thereby propelling the trafficking of ILC2s toward the lungs (89). Distinctly, ILC3s, which are the predominant producers of IL-22 and IL-17, are indispensable for the maintenance of intestinal barrier integrity (90). The gut microbiota directly modulates ILC3 function via metabolic byproducts (91). In the context of IBD, dysregulation of ILC3s precipitates barrier compromise; the aberrant inflammatory signals emanating from this dysfunction may systemically perturb the pulmonary microenvironment, thereby exacerbating susceptibility to COPD (92).

### 4.5 Intestinal barrier dysfunction and bacterial translocation

Gut and pulmonary inflammation reciprocally exacerbate one another via bidirectional mechanisms, perpetuating a vicious cycle of pathology. On one hand, compromised intestinal barrier integrity facilitates the systemic translocation of toxins, notably LPS, into the circulation. This triggers systemic inflammation via activation of the TLR4 pathway, thereby aggravating pre-existing pulmonary pathologies (93, 94). Reciprocally, pulmonary inflammation intensified by LPS leads to the downregulation of tight junction proteins, such as claudin-4, further attenuating intestinal barrier function (95). Concurrently, a sustained state of systemic inflammation and hypoxia acts via feedback mechanisms to compound pulmonary injury.

Clinically, plasma levels of Lipopolysaccharide-Binding Protein (LBP) and Zonulin are significantly elevated in COPD patients. Furthermore, these biomarkers exhibit a significant negative correlation with SarQoL scores (encompassing physical function, mental health, and cumulative scores). These findings furnish direct clinical evidence linking intestinal barrier dysregulation to COPD disease progression and adverse prognosis (96).

**Figure 2:**
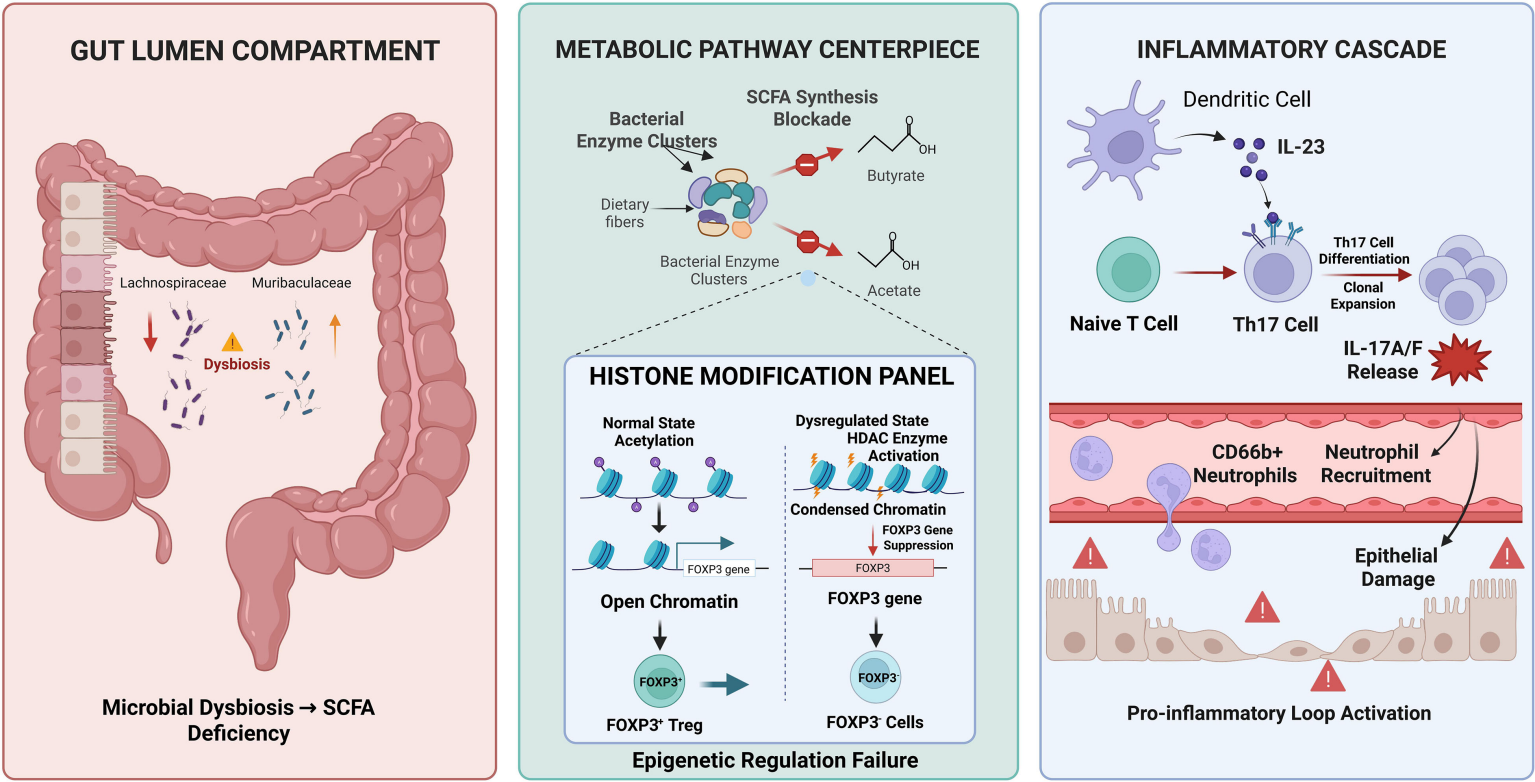
Immuno-metabolic dysregulation network. Depletion of SCFA-producing bacteria (e.g., *Lachnospiraceae*) reduces butyrate levels, impairing histone acetylation at the FOXP3 locus and inhibiting Treg differentiation. Concurrently, the IL-23/IL-17 axis is hyperactivated, driving Th17 expansion and neutrophilic damage. Treg, Regulatory T Cell; Th17, T Helper 17 Cell; IL, Interleukin; SCFA: Short-Chain Fatty Acid; HDAC, Histone Deacetylase; FOXP3, Forkhead Box P3; CD66b+, Cluster of Differentiation 66b Positive.

In summary, the crosstalk within the gut–lung axis is driven by a complex interplay of dysbiosis, immune dysregulation, and barrier failure. As illustrated in **Figure 2**, the loss of protective microbial metabolites (e.g., SCFAs) precipitates a cascade of epigenetic alterations and cytokine imbalances—most notably along the IL-23/Th17 axis, which ultimately disrupts mucosal integrity and perpetuates a systemic immuno-metabolic vicious cycle.

## 5 Molecular bridges: hypoxia, metabolites, and clinical confounders

### 5.1 Dual regulatory roles of hypoxia-inducible factors

Patients with COPD universally present with chronic hypoxemia, which leads to secondary ischemia and hypoxia within the intestinal mucosa. This consequently activates the HIF signaling pathway, a process characterized by significant isoform-specific regulation (97). HIF-1α predominantly mediates cellular adaptive responses to acute or moderate hypoxia, maintaining barrier integrity via mechanisms such as the upregulation of protective genes (98). Studies demonstrate that the deletion of HIF-1α in intestinal epithelial cells significantly exacerbates barrier injury induced by alcohol or pathogens, thereby facilitating LPS translocation (99).

However, under the chronic sustained hypoxic conditions associated with COPD, the HIF signaling pathway undergoes aberrant regulation (100). Animal models confirm that under states of chronic hypoxia and inflammation elicited by CS exposure, HIF-2α accumulates aberrantly (101). This accumulation drives pathological angiogenesis and inflammation, ultimately disrupting the mucosal barrier. Furthermore, circulating levels of vascular endothelial growth factor (VEGF), a biomarker of systemic hypoxia, correlate with both CS exposure and IBD activity, corroborating the holistic impact of the hypoxic environment on the gut-lung axis (102, 103).

While a unifying “Hypoxic Loop” is an attractive conceptual model to explain how respiratory pathophysiology mechanically enforces intestinal barrier disintegration, it generates specific and testable mechanistic predictions. At the mucosal level, this loop predicts the specific failure of intestinal tight junction complexes; indeed, hypoxia-driven signaling has been shown to precipitate the dysregulation and downregulation of critical barrier components, particularly Claudin-1, Claudin-4, and Zonula Occludens-1 (ZO-1) (104). Clinically, this structural failure should translate to a rise in circulating biomarkers of permeability and bacterial translocation. Corroborating these predictions, recent evidence demonstrates that plasma levels of LBP and Zonulin are significantly elevated in COPD patients (96). However, to rigorously establish this loop in humans, it is critical to acknowledge competing directions of causality. Severe systemic inflammation originating from active IBD can theoretically impair pulmonary microvascular endothelial function and worsen systemic oxygenation. Indeed, a significant proportion of IBD patients exhibit subclinical pulmonary abnormalities, including reduced carbon monoxide diffusing capacity (DLCO) and interstitial lung changes (5, 105), suggesting that gut-derived inflammation can bidirectionally drive pulmonary gas exchange impairment. Therefore, rather than a unidirectional driver, hypoxia likely establishes a reciprocal feedback loop between the gut and lungs.

Therapeutic interventions targeting this pathway show promise. Research indicates that CG-598, a HIF-prolyl hydroxylase inhibitor, effectively alleviates inflammation and augments intestinal barrier function in murine colitis models by stabilizing intestinal HIF-1α (106). Moreover, Hyperbaric Oxygen Therapy has been shown to attenuate neutrophil signal transducer and activator of transcription 3 activity, decrease the abundance of Akkermansia muciniphila, and elevate levels of mucin 2 and secondary bile acids. These effects synergistically ameliorate host intestinal inflammation and microbial dysbiosis, identifying intestinal mucosal hypoxia per se as an independent and intervenable therapeutic target (107).

### 5.2 Immunometabolic regulation by short-chain fatty acids

SCFAs, generated via the fermentation of dietary fiber by the gut microbiota, serve as pivotal immunometabolic mediators bridging diet, the microbiome, and pulmonary immunity (108). Mechanistically, butyrate, functioning as a potent histone deacetylase inhibitor, suppresses histone deacetylase activity to augment histone acetylation at the FOXP3 promoter locus (109, 110). This epigenetic modification fosters the differentiation and enhances the functional stability of Treg cells.

Gut-derived SCFAs traverse the systemic circulation to reach the lungs, where they exert pleiotropic immunomodulatory functions within distal tissues. Beyond serving as critical energy substrates for alveolar epithelial cells, they modulate dendritic cells to attenuate the priming of Th2-type immune responses. Concurrently, they directly suppress the production of IL-5 and IL-13 by ILC2s, thereby mitigating airway allergic inflammation (111–113).

In patients with COPD, a reduction in gut microbial diversity coupled with a significant depletion of SCFA-producing taxa directly compromises these distal anti-inflammatory and immunoregulatory mechanisms (114). Consequently, this metabolic deficit shifts the pulmonary microenvironment toward a sustained pro-inflammatory phenotype.

**Figure 3:**
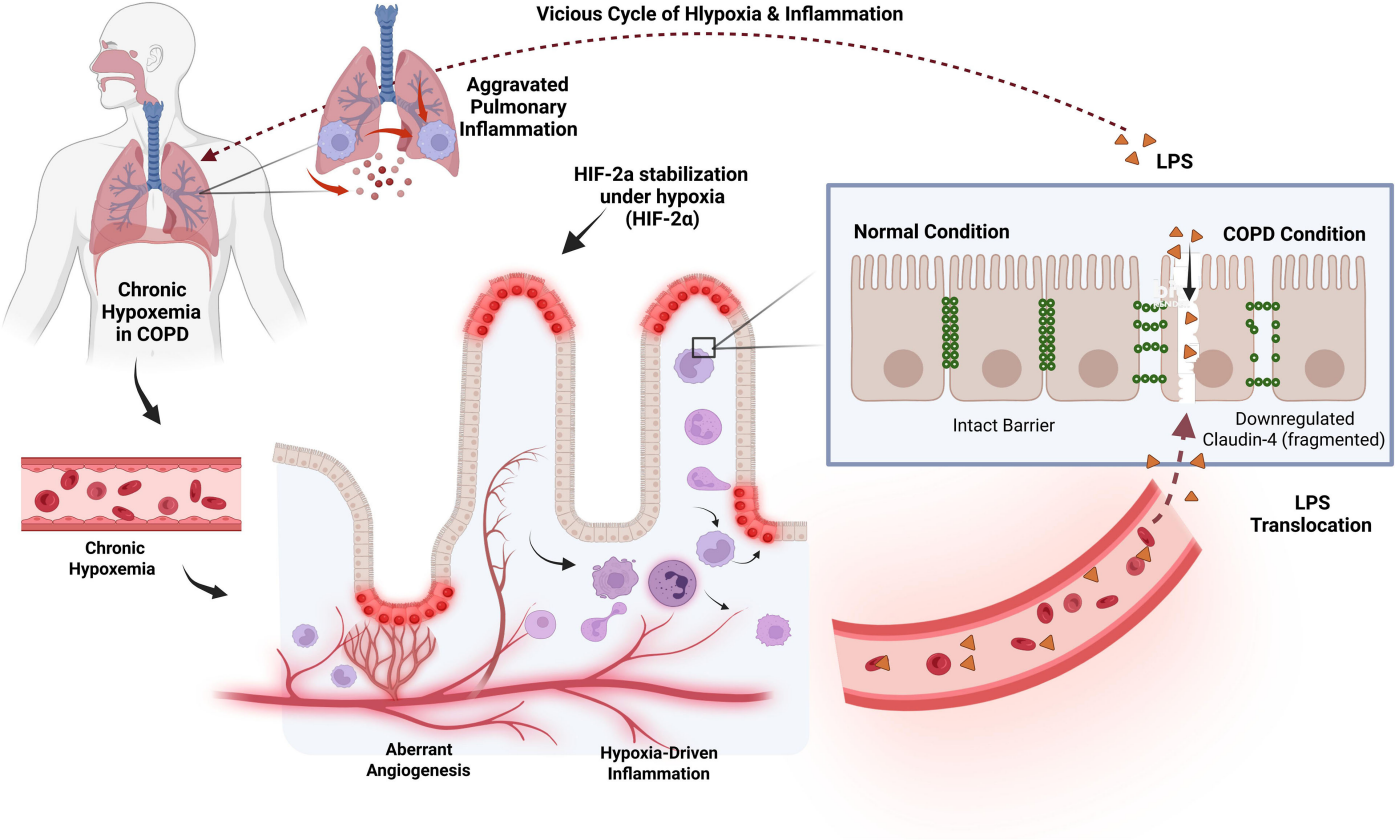
The hypoxic loop and barrier failure. COPD leads to systemic hypoxemia, causing intestinal ischemia. This triggers aberrant accumulation of HIF-2α in the gut epithelium, promoting pathological angiogenesis and inflammation. The resulting breakdown of tight junctions (e.g., Claudin-4) facilitates the translocation of LPS into the circulation (Leaky Gut), which feeds back to worsen pulmonary pathology. LPS, Lipopolysaccharide.

Ultimately, these molecular pathways converge to form a self-reinforcing loop of tissue injury. The systemic hypoxic environment characteristic of COPD not only directly compromises intestinal epithelial viability via aberrant HIF signaling but also synergizes with metabolic deficits to inhibit repair mechanisms. This distinct “Hypoxic Loop” (**Figure 3**) exemplifies how respiratory pathophysiology mechanically enforces intestinal barrier disintegration, thereby feeding back to exacerbate pulmonary inflammation.

### 5.3 Hidden axis: therapy as a confounder in gut-lung crosstalk

When interpreting observational data linking COPD and IBD, clinical therapies must be accounted for as profound confounders that independently reshape both the microbiome and systemic immunity.

Proton pump inhibitors (PPIs), widely prescribed for gastroesophageal reflux disease in COPD patients, reduce gastric acidity and profoundly decrease gut microbial diversity, facilitating ectopic colonization by oral/respiratory flora that mimics disease-associated dysbiosis (115, 116). Recurrent antibiotics for COPD exacerbations decimate intestinal obligate anaerobes, depleting SCFA-producing commensals and exacerbating Th17/Treg imbalance, potentially precipitating IBD flares independent of lung pathology (117).

Furthermore, corticosteroids (both systemic and inhaled), which are staples in managing acute flares for both diseases, have been shown to directly alter intestinal permeability and induce shifts in the microbiome composition, complicating the distinction between disease-driven and drug-driven barrier dysfunction (118). In addition, inhaled corticosteroids/long-acting β_2_ agonists (ICS/LABA) reduce the alpha diversity of the airway microbiota in COPD patients and induce more pronounced compositional shifts in the microbial community (119). Even oxygen therapy can exert unintended consequences on the gut. Therapeutic hyperoxia can disrupt the obligate anaerobic environment of the gut lumen, driving a dysbiotic shift towards facultative anaerobes like Proteobacteria, thereby triggering intestinal inflammation (120, 121).

Distinguishing true intrinsic gut-lung crosstalk from shared “iatrogenic systemic inflammation” remains a formidable challenge. Future associative studies must strictly control for medication burden and stratify cohorts by cumulative antibiotic/steroid exposure to ensure observed interactions reflect genuine pathology rather than pharmacological artifacts.

Importantly, the gut microbiome’s clinical relevance extends beyond confounding. Gut metabolic pathways have been directly associated with COPD and respiratory function, and microbial signatures predict immunotherapy outcomes in lung cancer, reinforcing the gut-lung axis as a therapeutic target (122–124).

## 6 Conclusion

The association between COPD and IBD transcends the realm of simple epidemiological comorbidity; at its core, it represents the shared manifestation of CMIS dysfunction across distinct anatomical sites. This complex pathophysiological process is underpinned by a shared genetic architecture, anatomical homology, and intricate systemic immune network interplay. Under homeostatic conditions, the gut and lungs maintain a continuous bidirectional dialogue via microbial metabolites, immune cell trafficking, and circulating cytokines. Genetic pleiotropy, as substantiated by GWAS, furnishes the molecular foundation for their shared susceptibility. While the CMIS confers the physiological capacity for cross-organ lymphocyte migration, in pathological states, the aberrant expression of homing receptors (such as CCR9) precipitates the ectopic dissemination of inflammation. At the microenvironmental level, gut dysbiosis and the consequent paucity of metabolites, superimposed by systemic hypoxia (HIF signaling) and cytokine storms, synergistically drive the reciprocal impairment of barrier function and a vicious cycle of inflammation.

Moving forward, establishing the cleanest “human proof-of-mechanism” for CMIS-driven cross-organ trafficking will require identifying organ-specific immune imprinting, such as the isolation of viable gut-imprinted α4β7+ T cells directly from the pulmonary interstitium of COPD patients, or conversely, lung-imprinted cells in the inflamed gut. Differentiating “shared systemic inflammation” from a true gut-lung axis in humans necessitates longitudinal discrimination of timing, targeted metabolite changes, and definitive organ-specific homing signatures.

Predicated on these mechanisms, future translational research must prioritize precision stratification and clinical translation. First, it is imperative to advance biomarker-based stratification of COPD patients to identify distinct subpopulations exhibiting a salient “gut phenotype”. These patients represent the potential prime candidates for gut-targeted interventions. Second, therapeutic strategies targeting shared nodal points, such as the IL-23 pathway or HIF signaling modulation, hold promise for achieving a unified therapeutic approach for these distinct pathologies. Consequently, subsequent research efforts should focus on elucidating the deep-seated molecular mechanisms of microbe-host interactions and validating the clinical efficacy of gut microbiota modulation or dual-target biologic therapies through rigorous prospective clinical trials.
